# Mothers' Response to Psychological Birth Trauma: A Qualitative Study

**DOI:** 10.5812/ircmj.10572

**Published:** 2013-10-05

**Authors:** Ziba Taghizadeh, Alireza Irajpour, Mohammad Arbabi

**Affiliations:** 1 School of Nursing and Midwifery, Isfahan University of Medical Sciences. Faculty Member of Nursing and Midwifery Care Research Center, Nursing and Midwifery School, Tehran University of Medical Sciences; 2 Nursing and midwifery Care Research Center, Isfahan University of Medical Sciences, Isfahan, IR Iran; 3 Psychiatry and psychology research center, Roozbeh hospital Department of Psychiatry,Tehran University of Medical Sciences, Tehran, IR Iran

**Keywords:** Traumatic Labor, Mother’s Response, Qualitative Research

## Abstract

**Background:**

Psychologically traumatic events can affect anybody, but consequences of psychological birth trauma for the mother are very profound, extensive and unforgettable. Furthermore, the mother’s response not only touches the mother, but also affects the child, the father and the society. The objective of this study was to explore the mothers’ response to psychological birth trauma.

**Objectives:**

Psychological birth trauma is a complex matter as the length of a women`s life and mother`s responds can be present through different psychological and physical ways. In this regard, the mothers suffer from its consequences, but they do not know what is going on? Mothers are getting worse every day by “the silent effects of the psychological phenomena”.

**Materials & Methods:**

This qualitative study was conducted on 23 mothers with psychological birth trauma experience, who were recruited from health centers of the capital and one of the metropolises of Iran. Their interviews were transcribed verbatim and analyzed by the content analysis method.

**Results:**

Three themes were extracted from the data: impact on health, changes in mother`s roles, and changes decision making ability. Several categories and sub-categories also emerged from the data (physical and psychological problems, bonding with the child, relationship with husband, social role, cesarean request and psychological inability to have another child).

**Conclusions:**

By considering the mothers` responses to traumatic labor, which endangers the health of the child as well as that of the mother and impairs their familial and social relationships, midwives should notice the consequences of psychological birth trauma in order to plan supportive and timely interventions.

## 1. Background

Pregnancy and childbirth can lead women down two strikingly different paths. One path can get off to a very good start to motherhood, whereas in case of traumatic labor, the other path can lead them to a very poor start in their relationship with the child and husband, as well as to psychological problems (1). It may also cause some problems in mothers` social lives. Whereas birth is a powerful experience, when it is traumatic, its impacts can be considerable and unforgettable (2). Psychological birth trauma (PBT), also referred to as traumatic childbirth (TB), is a situation in which the woman has suffered distress as a result of injury to herself and her baby, or pain or sorrow, which is in such a magnitude that it may prone the mother to a traumatic condition, with a prolonged psychological and/or physical effect (3).

It has long been recognized that some women, following a traumatic childbirth, go on to develop some psychological disorders (4). In this connection, Hofberg and Ward believed although pregnancy and childbirth are often desired by women, it is not uncommon to experience some degree of anxiety (5). Gamble also found a high prevalence of postpartum depression and trauma symptoms occurring after childbirth (6). The prevalence of psychological birth trauma at a rate between 20 to 30% has been reported by different authors in different countries (6-8). Creedy et al. also reported that one out of every three births can lead mothers to psychological birth trauma. Feeling out of control, depressed, anxious and post-traumatic stress disorders are the consequences of psychological birth trauma (9, 10). In one study, 1.9% of women perceived birth as traumatic and progressed to post-traumatic stress disorder (8). In addition, about 13% of all women will experience an episode of postnatal depression (1).

Psychological birth trauma can also hurt family relationships (11), reduce the lactation period (12) and, in the long run, the children of these mothers end up with emotional, cognitive and behavioral disorders (13, 14). Postnatal depression may adversely influence infant and child development, as well as having potential negative consequences on women and their families (1, 15). Moreover physical disorders such as those accompanied by excessive fatigue, vital exhaustion, and reduction in functional capacity can be caused by depression following psychological birth trauma (15). Four women in one study suffered from long-term physical consequences from their birth, such as severe pain (16). Furthermore, requesting an elective cesarean birth implies a high level of anxiety about childbirth. However, elective cesarean section does not ‘‘cure’’ the fear of childbirth. Thus, when a pregnant woman requests an elective cesarean section that is not medically endorsed and counseling is recommended (2).

Regarding psychological inability to have another child, the most significant finding of one study was that women with a negative experience from their first birth decided not to have another child or considered a longer interval before the second birth (17). Turning to the mother`s roles, different bonding styles with the child were reported by Nicholls and Ayers which seemed split between “overprotective/anxious bonds and avoidant/rejecting bonds”; in the former, some women reported acting out the mothering role (delayed onset of emotional attachment) (18). Also, in one study, initial feelings of rejecting their neonate were reported by the majority of the participants (16). In some cases, women believed that their ability to bond with their children had been affected by the negative childbirth experiences (19). With respect to the relationship with their husband, there is some evidence around sexual avoidance and fear of childbirth. During the first year, following childbirth, some women could not have a sexual relationship with their partners and had a cold and distant sexual behavior with them (4). In addition, some men and women perceived difficulty with intimacy due to birth (18). In one study all women were under pressure from their relationship with their partners due to the traumatic birth experience (16).

## 2. Objectives

Psychological birth trauma is a complex matter as the length of a women`s life and mother`s responds can be present through different psychological and physical ways. In this regard, the mothers suffer from its consequences, but they do not know what is going on? Mothers are getting worse every day by “the silent effects of the psychological phenomena”. However, true figure of psychological birth trauma is unknown, as many women do not seek help. Women suffer more than men according to surveys in Iran and other countries (20, 21). Misdiagnosis, delay, non-specific treatments and lack of follow up have constituted a typical care pathway for depressed people throughout the world. One reason may be that the explanatory models of clinicians differ from those of patients in their own culture (22). Furthermore, in many countries the cost of psychological treatment is very high, not only in terms of social but also economic issues. For example referring to a psychologist is a “social stigma” for the mothers and it costs them a lot of money. Knowledge about psychological birth trauma is restricted; moreover the mother`s response as an ambiguous concept varies in different cultural, social, economic and environmental contexts, so this qualitative study tries to describe the mothers' response to psychological birth trauma.

## 3. Materials and Methods

In order to understand mother`s response to traumatic birth, a qualitative study with content analysis approach was adopted. Content analysis as a research method is a systematic and objective means to describe phenomena (23). Krippendorff believed that content analysis is a research method for making replicable and valid inferences from data to the context of the data, with the purpose of contributing knowledge, new insights and a representation of facts: to achieve a condensed and broad description of the phenomenon (23, 24). In this study, inclusion criteria comprised of an experience of psychological birth trauma assessed using the revised fourth edition of the standard psychiatric questionnaire DSM, ability to speak and understand the Persian language, and exclusion criteria consisted of any proven post-trauma stress or psychotic disorders. We did not exclude any mothers during sampling. In order to achieve maximum variation of participants and to allow transferability of research findings, age, number of pregnancies, educational level, economic, social and cultural status, employment status and mode of delivery were considered in the process of purposive sampling.

### 3.1. Data Collection Procedures

Approval to conduct the study was obtained from the institution’s ethics committee. The participants were informed about the aim of the study and signed a written informed consent for the taped recorded interview. They were assured of their privacy and also informed that they could withdraw from the study without consequences. The interviews were conducted at a convenient time and place for the participants. The mean time of interviews was about 60 minutes. Interviews were conducted until saturation was achieved.

### 3.2. Data analysis

The participants were the mothers from Tehran and Isfahan health centers, who experienced traumatic childbirth, recruited from September 2011 to February 2012. Participants were aged between 18 to 50 years and their educational statuses ranged from secondary school to doctoral degree and were mostly housewives. The Reproductive characteristics of participants are shown in Table 1. 

**Table 1.** Reproductive Characteristics of Participants

Reproductive Variables	Percentage (Frequency)
**Parity**	
Nulliparous	11 (47.82)
Multiparous	12 (52.17)
**Types of delivery**	
Vaginal delivery	6 (26.08)
Caesarean section	17 (73.91)
**History of infertility**	
Yes	12 (52.17)
No	11 (47.82)
**Complications of pregnancy**	
Yes	14 (60.86)
No	9 (39.13)
**Wanted or unwanted pregnancy**	
Desirable	15 (65.21)
Undesirable	8 (34.78)
**Wanted or unwanted gender of baby**	
Desirable	19 (82.60)
Undesirable	4 (17.39)
**Types of hospital**	
State	17 (73.91)
Private	6 (26.08)

Initial interviews were conducted with 94 mothers to determine their eligibility; any mother, who was suspected to have experienced PBT, underwent a semi-structured interview. After each interview verbatim transcriptions were used to illustrate each finding. Thereby, twenty-three mothers were identified to be eligible. Next, responses were coded using content analysis into three major categories.

### 3.3. Mothers’ Responses to Psychological Birth Trauma

The result of the interview with mothers showed that, if childbirth makes maternal psychological trauma, it would vitiate their social rapport, leading them to live an isolated life and ultimately a manifestation of their psychological disorders. Likewise, mother’s decision-making power for her future reproductive life would be affected by this trauma. Interpersonal communication problems and uncertainty in decision-making about her reproductive life thus constituted the theme, “mothers’ responses to psychological birth trauma”. There was a widespread disrupt relationship between the mother and her husband, children and relatives. The participant mothers, who suffer from psychological birth trauma, demonstrated similar reactions through different ways. The relationship gulf between mother and her child, challenging marital life, and social conflict are incorporated in the subcategory of communicative problems as the failure to a subsequent pregnancy; if the subsequent pregnancy takes place, resorting to caesarean section is incorporated to sub-category of uncertainty in decision-making. Table 2, represent the theme, “mothers’ responses to psychological birth trauma”, categories, sub-categories and the contracted meaning unit. The responses of participating mothers in this psychological birth trauma study were as follows.

**Table 2.** Summary of the Theme “Mothers’ Responses to Psychological Birth Trauma”, Categories, Sub-Categories and Contracted Meaning Unit

Theme	Categories	Sub-Categories	Contracted Meaning Unit
Mothers’ responses to psychological birth trauma	Communicative problems	The relationship gulf between mother and child	Feeble attachment to child
			Unable to fulfill her duty as a mother
			Reluctant to feed him on her breast milk
		Demanding marital life	Emotional and sensitive apathy
			Uninterested to have sexual intercourse
		Social conflict	Social escape
			Social conflict
	Uncertainty in decision-making	Failed to have a later pregnancy	
		resorting to caesarean section in case of a later pregnancy	

#### 3.3.1. Relationship Gulf Between Mother and her Child

The sub-category of relationship gulf was constituted by the two concepts; mother’s feeble attachment to her baby and inability to fulfill the maternal role. The communicative gulf between the mother and her child, which is due to psychological trauma, did not go away, even with time. One of the mothers said about her relationship with her child; “I didn’t love my baby during the first three to four months as everybody came to kiss her and treated her with a sense of love, and the only person who didn’t have these feelings for her was me, I never thought she was my child, I don’t know why, I could only feed her my breast milk and there was no other feeling that I could have for her. When she was inside my belly, I liked her but this feeling went away once I gave birth to her. Since the childbirth, I don’t look like a normal human being”. A mother who had experienced pregnancy 8 months ago said “I wasn’t happy at all as evident in the video filmed in the delivery ward of the hospital, I didn’t feel anything special when my baby was brought close to my face. I felt nothing so I pulled my face away from her, it meant that I didn’t have any pleasant feeling about this, at this point I don’t have any feelings for my baby, I have no idea, maybe it will be fine when she grows up, and she can walk or talk to me”.

In case there was no attachment between the mother and the child, the mother’s role would be ruined. A young mother, after 3 years from her delivery, said about her first and only child: “such hollow feeling drove me to offer my child to my aunt. My husband, asked: Are you sure yet that you really want to give our child to your aunt ? And I replied to him, “No, I can’t”. But this was a lie, because I wanted to do so. I wanted to fix this relationship, really; but I couldn’t. I’m trying but it doesn’t work. I am experiencing hollow feelings about my child. I couldn’t take care of her. Such relationship gulf would disrupt the breastfeeding process, too. One of the mothers said about her first pregnancy (at the time of this interview, she had two kids): “at this point, I wish my first child could go back to infancy and I could feed her my breast-milk, kindly. How hard it was to breastfed her.”

#### 3.3.2. Demanding Marital Life

Sensitive and emotional apathy along with a reluctance to have sexual relationship constitutes the “demanding marital life” class. Emotional and sexual relationships of most mothers are subject to be ruined by the psychological birth trauma. One mother, regarding this matter, said: “my anxiety affected my marital relationship. We became apart; our love decreased. It affected our life directly.” One of the mothers said: “our emotional relationship decreased after childbirth. I lost my sex drive. I don’t know what is my problem? I was damaged. It’s been four months since I have no interest to have sex with him. After this period, our relationship was interrupted. There was no pattern. At this point in time, I think we are going to reach that pattern, it’s been three years since I have had the delivery, and we are going back to the previous pattern, little by little”.

#### 3.3.3. Social Conflict

The mothers’ relationships with their relatives were summed up based on two battling and struggling perspectives under the sub-category of social conflict. Following the psychological birth trauma, some mothers displayed aggressive behaviors towards their relatives, as others preferred to remain shy and isolated. The response, which make up the theme of social escape was as follows; “my husband knows, it means that he realized the change in my demeanor, I used to laugh a lot, happy, funny, but I spoke less and slept much, I hardly made an effort to do anything”. Another mother said “I was much into staying under the duvet. People’s chatter annoyed me. Baby’s voice vexed me, other’s voice made me sick”. The titles, wall and submissive lamb, which represent an inactive person, were used by two mothers as metaphors for their feelings and responses after the psychological birth trauma. A participant mother said “I had forgotten everybody, nobody mattered to me, when I was in a normal state, I cared about the person who came to see me, I especially liked guests, and I had a lot of things to do. However, I had never welcomed any guest coming to my house after the delivery, what else I can say! I looked as if I was a wall and I didn’t have the same feelings I had before”. Another mother said “I felt as if I was a little lamb, when I went to the delivery ward, it seemed to me that I had handed everything over to them so that they could make any decision they wanted. As far as I didn’t realize anything, I had to give in to what they wanted; I couldn’t take it any longer”. Some mother participants sought to get into a fight. One of them said: “I didn’t allow doctors to examine me as they insisted to do so. I said I don’t like to be touched, I felt weaker in spirit than before because I couldn’t stand it”. Another one said: “once they intended to do something for me, I didn’t let them go ahead. It wasn’t my fault. I was scared to death”.

#### 3.3.4. Uncertainty of Decision-Making

The failure to have a later pregnancy and resorting to caesarean section in case of a later pregnancy, which in turn constituted the sub-category of uncertainty in decision-making, were manifested in the other mothers’ responses to the psychological trauma; these consequences embody in the maternal future reproductive life. One of the mother participants, who expressed her lack of interest in having the second child, said: “I don’t think of having another baby; I don’t, because I don’t want the hard time I had gone through to recur”, another participant said: “the one thing that has always annoyed me and put an obstacle on my way forward. Due to this, pregnancy process annoyed me. Even though it gave me a good result and I have a healthy kid and he is alive, it made me upset a lot. Such psychic harassment is with me and that's why I prefer to have only one kid". Another mother, who had only one child and went through the delivery eight years ago, said: "that kind of fear is lingering in my head. This is a reason that made me refuse a later pregnancy, as the same procedure would happen to me, things that I’m really afraid of, why do I have to go through it again?” Some mothers desperately used caesarean in order to run away from the delivery. One of the mothers said: “this delivery made me feel frightened, otherwise I would like to have another baby, I can’t stand seeing the delivery ward again, its pains, I don’t want it. If the situation allows for an easy delivery, I wouldn’t dislike having another baby. But when it comes to the delivery ward, I would say no. By the way, if I once again get pregnant, I would definitely have a caesarean”. Another one also said that “what I can say about the delivery!? It was horrible. People told me it is literally difficult to the extent that you feel you are dying. That’s way I preferred not to give birth to my child naturally, instead I chose to have a caesarean. This was the very reason that drove me to have a caesarean, that’s why I got frightened. Now, as I went through the delivery by myself, I will never ever have a normal delivery.”

## 4. Results

Three themes were extracted from the data: impact on health, changes in mother’s roles, and changes decision making ability. Several categories and sub-categories also emerged from the data (physical and psychological problems, bonding with the child, relationship with husband, social role, cesarean request and psychological inability to have another child).

## 5. Discussion

This study explored the mothers' response to psychological birth trauma. On the whole, results of our study revealed that psychological birth trauma as an umbrella can shadow on the mother`s life through changing the mother`s roles, ability of their decision making and also impact on their health. All dimensions are discussed as followed.

### 5.1. Impact on Mother`s Health

In the developing world, women often do not have adequate antenatal care and may be psychologically unprepared to face hospitalization and procedures related to labor (25). So although the vast majority of women recover quickly after childbirth, for some of them prolonged suffering remains, that impacts on their lives and on the family members` lives (26). In our study, the responses of the mothers to psychological birth trauma consist of some psychological disorders such as mood disorders, thought disorder, perception and also physiological disorders. Majority of the participants cried even from thinking about the birth process. They had a profound grief and suffered from depression, anxiety, PTSD and even one of them suffered from psychosis. Beck and Ayers believed that psychological morbidity is common during women`s childbearing periods and some women perceived childbirth as a traumatic event which led them to experience posttraumatic stress reaction (27-29), anxiety and depression (30) and forced the mother to have an extra visit after physical symptoms (31). Some studies from Australia showed that 26% of pregnant women had fatigue and sleep deprivation due to strong fear of childbirth (32, 33).

### 5.2. Changes in Mother`s Roles

Mother’s interaction in this study was affected by the fear of childbirth. Nonetheless, experiencing psychological trauma during childbirth, irrespective of development to posttraumatic stress disorder, can have a negative impact on the mother’s psychological functioning (8), such as difficulty in relationship with the husband and bonding and long-term attachment problems with the child (31, 34, 35) and led the mothers to a painful isolation from the world of motherhood (36). Women’s sexuality complications are an other outcome from fear of childbirth (34). In this regard, three women reported a lack of understanding from their partners (16). Our study`s results were supported by previous findings that experiencing a traumatic birth can end to some problems in bonding with the child or in relationship with the husband. Allen found an impact of psychological birth trauma on the mother`s relationship with others (37). In our study, some of the participants could not follow their health care providers` order and also they could not have sociability with their families or their friends whereas other studies did not support these findings.

### 5.3. Changes in Ability of Decision Making

In this study due to fear of childbirth, mothers had lost their decision-making power. In agreement to this finding, several studies showed that when the fear of childbirth, manifested as stress symptoms, it affected mother`s everyday life and resulted in a wish to avoid pregnancy and childbirth (5, 37-39). Results of a recent Swedish study by Skari at el. showed that a negative birth experience is related to a reduced probability of having a subsequent child (1). Unfortunately research about psychological birth trauma is scant, and most has focused on the development of posttraumatic stress disorder after childbirth, which appears to be a relatively rare phenomenon; however, the experience of trauma may be much more prevalent (8). Beck and Watson (2010) stated that women experience an intense fear following a traumatic birth (7). Sever fear of childbirth affects the daily life of 6% of pregnant women and approximately 10% of Swedish pregnant women suffer from extraordinarily fear of childbirth (37). Eleven percent (11%) of Swedish women (40), 5.3% of Swiss women (41), and 78% of Finnish women (42) suffered from childbirth due to the impact of intense fear. In this regard twenty-two women believed that their whole care during childbirth had been mismanaged (19).

In order to cover the whole spectrum of psychological and behavioral responses, Skari believed that social role functioning, and psychological distress, such as anxiety and depressive symptoms, should be measured (1). Hall believed that we should find the way to diminish anxiety followed by traumatic birth (33). Regarding the results the authors believed that attention to the mental health of women in childbirth and its consequences is the main strong point of this study. Also, interview with families, especially with the husband could be useful, but in this study, interviews were conducted only with the mothers. This is the weak point of the study. It can be explored in further researches.

Results of the study showed that all aspects of women’s health are endangered due to traumatic childbirth. According to the matter that women's health not only forms half of the population's health, but also effects the health of the whole society, it seems necessary to psychologically prepare mothers to face hospitalization and procedures related to childbirth by designing innovative approaches and establishing friendly centers for protection of mothers` health in physical, psychological and social aspects.

## References

[1] Skari H, Skreden M, Malt UF, Dalholt M, Ostensen AB, Egeland T (2002). Comparative levels of psychological distress, stress symptoms, depression and anxiety after childbirth—a prospective population‐based study of mothers and fathers.. BJOG: Int J Obstet Gynaecol..

[2] Gardner PS (2003). Previous traumatic birth: An impetus for requested cesarean birth.. J Perinat Educ..

[3] Moyzakitis W (2004). Exploring women's descriptions of distress and/or trauma in childbirth from a feminist perspective.. Evidence Based Midwifery..

[4] Bailham D, Joseph S (2003). Post-traumatic stress following childbirth: a review of the emerging literature and directions for research and practice.. Psychology, Health & Medicine..

[5] Hofberg K, Ward MR (2003). Fear of pregnancy and childbirth.. Postgrad Med J..

[6] Gamble J, Creedy D, Moyle W, Webster J, McAllister M, Dickson P (2005). Effectiveness of a counseling intervention after a traumatic childbirth: a randomized controlled trial.. Birth..

[7] Beck CT, Watson S (2010). Subsequent childbirth after a previous traumatic birth.. Nurs Res..

[8] Soet JE, Brack GA, DiIorio C (2003). Prevalence and predictors of women's experience of psychological trauma during childbirth.. Birth..

[9] Gamble JA, Creedy DK, Webster J, Moyle W (2002). A review of the literature on debriefing or non-directive counselling to prevent postpartum emotional distress.. Midwifery..

[10] Harris R, Ayers S (2012). What makes labour and birth traumatic? A survey of intrapartum ‘hotspots’..

[11] Beck CT (2006). The anniversary of birth trauma: Failure to rescue.. Nurs Res..

[12] Henderson JJ, Evans SF, Straton JAY, Priest SR, Hagan R (2003). Impact of postnatal depression on breastfeeding duration.. Birth..

[13] Galler JR, Ramsey FC, Harrison RH, Taylor J, Cumberbatch G, Forde V (2004). Postpartum maternal moods and infant size predict performance on a national high school entrance examination.. J Child Psychol and Psychiat..

[14] Righetti-Veltema M, Bousquet A, Manzano J (2003). Impact of postpartum depressive symptoms on mother and her 18-month-old infant.. European child & adolescent psychiatry..

[15] Fisher JRW, Feekery CJ, Rowe-Murray HJ (2002). Nature, severity and correlates of psychological distress in women admitted to a private mother–baby unit.. J Paediatr Child Health..

[16] Ayers S, Eagle A, Waring H (2006). The effects of childbirth-related post-traumatic stress disorder on women and their relationships: A qualitative study.. Psychol Health Med..

[17] Gottvall K, Waldenström U (2002). Does a traumatic birth experience have an impact on future reproduction?. BJOG: Int Obstet Gynaecol..

[18] Nicholls K, Ayers S (2007). Childbirth‐related post‐traumatic stress disorder in couples: A qualitative study.. Brit J health Psychol..

[19] Porter M, Van Teijlingen E, Chi Ying Yip L, Bhattacharya S (2007). Satisfaction with Cesarean Section: Qualitative Analysis of Open‐Ended Questions in a Large Postal Survey.. Birth..

[20] Assadi SM, Nakhaei MR, Najafi F, Fazel S (2007). Mental health in three generations of Iranian medical students and doctors.. Soc Psychiatry Psychiat Epidemiol..

[21] Dejman M (2010). Cultural explanatory model of depression among Iranian women in three ethnic groups (Fars, Kurds and Turks)..

[22] Dejman M, Forouzan A, Assari S, Farahani MA, MalekAfzali H, Rostami AF (2009). Clinicians’ view of experience of assessing and following up depression among women in IR Iran.. World Cult Psychiatry Res Rev..

[23] Elo S, Kyngäs H (2008). The qualitative content analysis process.. J Adv Nurs..

[24] Herring SC (2010). Web content analysis: Expanding the paradigm.. Int Hand Internet Res..

[25] Chandra PS (2009). Part Introduction.. Contemp Top Women's Mental Health..

[26] Reynolds JL (1997). Post-traumatic stress disorder after childbirth: the phenomenon of traumatic birth.. Can Med Assoc J..

[27] Ayers S (2007). Thoughts and emotions during traumatic birth: A qualitative study.. Birth..

[28] Beck CT (2004). Birth trauma: in the eye of the beholder.. Nurs Res..

[29] Elmir R, Schmied V, Wilkes L, Jackson D (2010). Women’s perceptions and experiences of a traumatic birth: a meta‐ethnography.. J Adv Nurs..

[30] Laursen M, Hedegaard M, Johansen C (2008). Fear of childbirth: predictors and temporal changes among nulliparous women in the Danish National Birth Cohort.. BJOG: Int Obstet Gynaecol..

[31] Poikkeus P, Saisto T, Unkila-Kallio L, Punamaki RL, Repokari L, Vilska S (2006). Fear of childbirth and pregnancy-related anxiety in women conceiving with assisted reproduction.. Obstet Gynecol..

[32] Fenwick J, Gamble J, Nathan E, Bayes S, Hauck Y (2009). Pre‐and postpartum levels of childbirth fear and the relationship to birth outcomes in a cohort of Australian women.. J Clin Nurs..

[33] Hall WA, Hauck YL, Carty EM, Hutton EK, Fenwick J, Stoll K (2009). Childbirth fear, anxiety, fatigue, and sleep deprivation in pregnant women.. J Obstet Gynecol Neonatal Nurs..

[34] Hofberg K, Ward MR (2004). Fear of childbirth, tocophobia, and mental health in mothers: the obstetric-psychiatric interface.. Clin Obstet and Gynecol..

[35] Nieminen K, Stephansson O, Ryding EL (2009). Women's fear of childbirth and preference for cesarean section–a cross‐sectional study at various stages of pregnancy in Sweden.. Acta obstetricia et gynecologica Scandinavica..

[36] Beck CT (2004). Post-traumatic stress disorder due to childbirth: the aftermath.. Nurs Res..

[37] Allen S (1998). A qualitative analysis of the process, mediating variables and impact of traumatic childbirth.. J Reprod Infant Psychol..

[38] Melender HL (2002). Fears and coping strategies associated with pregnancy and childbirth in Finland.. J Midwifery Women Health..

[39] Wiklund I, Edman G, Ryding EL, Andolf E (2008). Expectation and experiences of childbirth in primiparae with caesarean section.. BJOG: Int J Obstet Gynaecol..

[40] Waldenström U, Hildingsson I, Ryding EL (2006). Antenatal fear of childbirth and its association with subsequent caesarean section and experience of childbirth.. BJOG: An International Journal of Obstetrics & Gynaecology..

[41] Nilsson C, Lundgren I (2009). Women's lived experience of fear of childbirth.. Midwifery..

[42] Melender HL, Lauri S (2002). Experiences of security associated with pregnancy and childbirth: A study of pregnant women.. Int Nurs Pract..

